# Subdural and Cerebellar Hematomas Which Developed after Spinal Surgery: A Case Report and Review of the Literature

**DOI:** 10.1155/2013/431261

**Published:** 2013-07-11

**Authors:** Ufuk Utku, Sibel Güler, Erol Yalnız, Ercüment Ünlü

**Affiliations:** ^1^Department of Neurology, Faculty of Medicine, Trakya University, 22030 Edirne, Turkey; ^2^Department of Orthopedics and Traumatology, Special Ekol Hospital, Edirne, Turkey; ^3^Department of Radiology, Faculty of Medicine, Trakya University, 22030 Edirne, Turkey

## Abstract

Cerebellar hemorrhage following a spinal surgery is extremely rare; however, considering the localization, it can cause major clinical manifestations. While it is considered that these types of bleedings occur secondary to a venous infarct, the pathogenesis is still unclear. A 57-year-old male patient who underwent a laminectomy by exposing T12-L5 and had pedicle screws placed for ankylosing spondylitis developed a CSF leak due to a 2 mm dural tear. A hemorrhage with parallel streaks on the left cerebellar hemisphere was seen in CT scan, and a thin subdural hematoma at right frontotemporal region was seen on cranial MRI, performed after the patient developed intense headache, nausea, vomiting, and stiff neck in the early postoperative period. In this paper, a case of cerebellar and subdural hematomas following a spinal surgery is discussed with its clinical and radiologic findings.

## 1. Introduction

It is reported that the rate of dural tear during spinal surgery is 1%–17%, and while it can be associated with surgical technique, it can also be related to patient factors like “thin dura” or “dural adhesions” [1]. Intracranial hypotension resulting from an excessive CSF leak due to a dural tear causes postural headaches, photophobia, and altered mental status. Usually this condition improves with bed rest and fluid supply for a few days. Another rare complication caused by CSF leak during a spinal surgery is intracranial hemorrhage. In the presence of neurological deterioration in cases of intraoperative dural tear and CSF leak, a careful evaluation must be done in terms of cerebellar hemorrhage. While most of these bleedings occur in cerebellar localization, it can occur rarely in subdural or epidural spaces [1–3]. In this paper, a case of cerebellar and subdural hematomas following a spinal surgery is discussed with its clinical and radiologic findings.

## 2. Case

**Figure 1:**
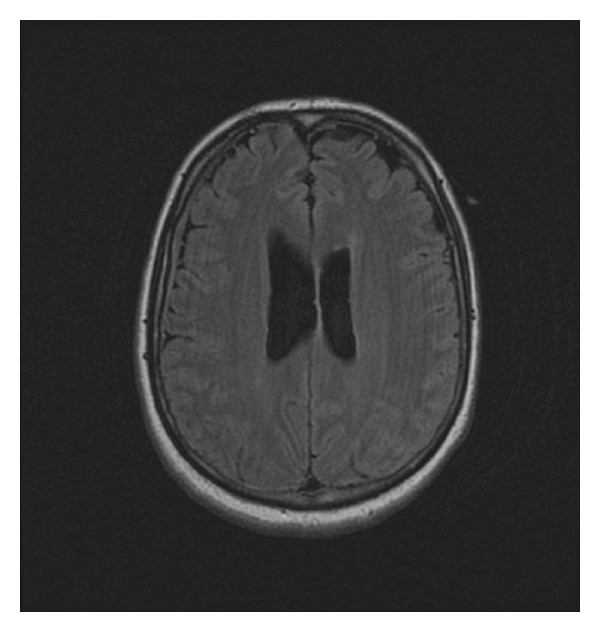
Cranial MRI image showing thin subdural hematoma in the right frontotemporal region.

**Figure 2:**
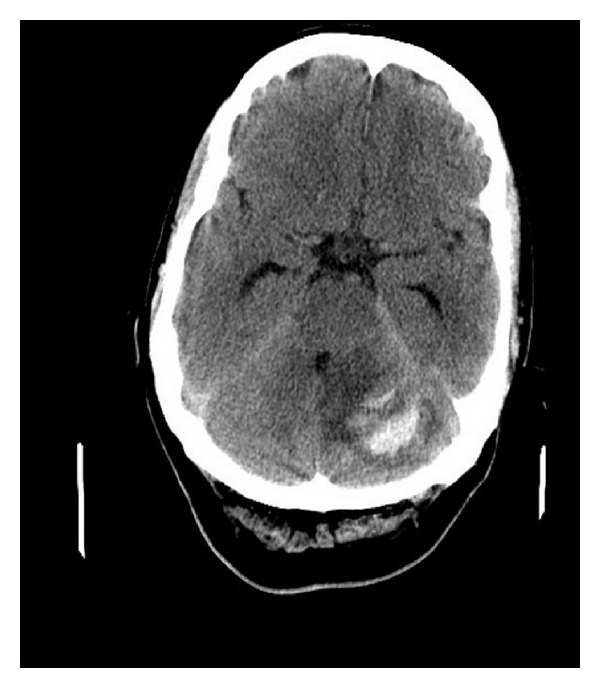
Cranial BT image showing bleeding at the site of left cerebellar hemisphere with streaky pattern (zebra sign).

**Figure 3:**
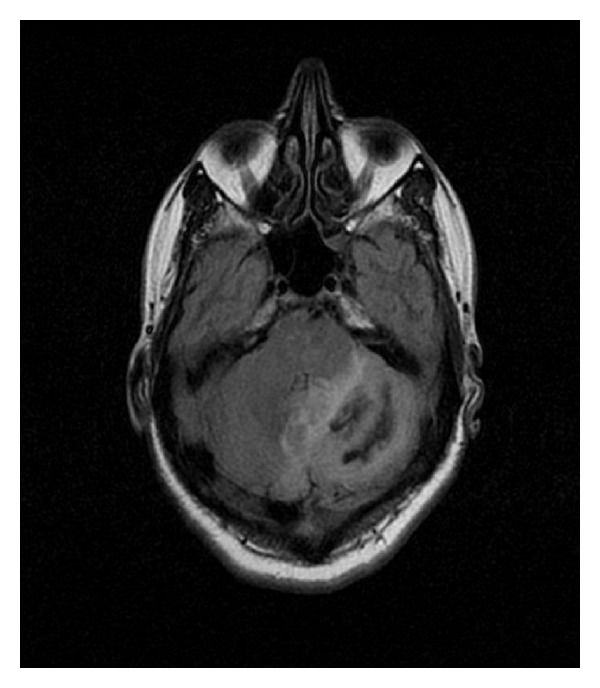
Cranial FLAIR enhancement MRI image showing left cerebellar hemorrhage.

A 57-year-old male patient who underwent a laminectomy by exposing T12-L5 and had pedicle screws placed for ankylosing spondylitis developed a CSF leak due to a 2 mm dural tear. A hemorrhage with parallel streak on the left cerebellar hemisphere was seen in CT scan, and a thin subdural hematoma at right frontotemporal region was seen on cranial MRI, performed after the patient developed intense headache, nausea, vomiting, and stiff neck in the early postoperative period (Figures 1, 2, and 3). Both cranial MRI angiography and venography were normal. Patient's coagulation tests were normal as well as his blood pressure. Patient was given fluid replacement, antiedema therapy (4 mg dexamethasone every six hours), and bed rest. Symptoms improved rapidly with the treatment. Also a follow-up CT scan showed the regression of the hematoma.

## 3. Discussion

The case of a cerebellar hematoma following a spinal surgery was first reported by Chadduck in 1981, and 30 more cases were reported so far in the literature [4]. Dural tear during spinal surgery is not common, and its incidence is reported as approximately 0.3%–5.9% [5]. The cerebellar hematoma occurs probably due to downward displacement of the cerebellum and stretch of the superior vermian vein, leading to venous hemorrhagic infarct [1, 2]. Yacubian et al. suggested that brain dislocation resulting from intraoperative excessive cerebrospinal fluid drainage is a possible mechanism [6]. Göbet et al. reported that the pathophysiology of the cerebellar hemorrhage following a spinal surgery is the mechanical stress caused by CSF loss after surgery, resulting in traction and tearing of cerebellar veins [7]. It is stated that factors like arterial hypertension, anticoagulant therapy, aneurism, and arteriovenous malformation can cause cerebellar hemorrhage. In our case, there was not a history of hypertension, coagulopathy, or an evidence in MR angiography suggesting an arteriovenous malformation.

Friedman et al. reported that cerebellar hemorrhage usually occurs at the site of vermis and superior segment of the cerebellar hemispheres [8]. In our case, the cerebellar hemorrhage was observed in the inferior and superior parts of the left cerebellar hemisphere. As in most of the cases, the streaky bleeding pattern named “zebra sign” was seen in our case too, and it is thought to be due to blood spreading in the cerebellar sulci [9]. Subdural hematoma seen in our case is a rare condition and it was only reported in 7 cases [10]. The mechanism of subdural hematoma is explained by tearing of stretched bridging veins due to downward dislocation of the brain [2, 11]. Interestingly, in our case, subdural hematoma was seen along with cerebellar hemorrhage after the spinal surgery.

Most of these cases in the literature have shown a rapid improvement with medical treatment and bed rest, but rare cases like brain stem compression and hydrocephaly needed a decompression therapy [12]. In our case, clinical symptoms improved rapidly with medical treatment.

Cerebellar hemorrhage might occur due to a CSF leak during or after any type of spinal surgery with dural tear or intradural manipulation [13]. To our knowledge, the case we presented is the only case where both cerebellar and subdural hematomas can be seen, thus making it worth presenting.

## References

[1] Beier AD, Soo TM, Claybrooks R (2009). Subdural hematoma after microdiscectomy: a case report and review of the literature. *Spine Journal*.

[2] Burkhard PR, Duff JM (2000). Bilateral subdural hematomas following routine lumbar diskectomy. *Headache*.

[3] Surash S, Bhargava D, Tyagi A (2009). Bilateral extradural hematoma formation following excision of a thoracic intradural lesion: case report. *Journal of Neurosurgery*.

[4] Chadduck WM (1981). Cerebellar hemorrhage complicating cervical laminectomy. *Neurosurgery*.

[5] Andrews RT, Koci TM (1995). Cerebellar herniation and infarction as a complication of an occult postoperative lumbar dural defect. *American Journal of Neuroradiology*.

[6] Yacubian EM, de Andrade MM, Jorge CL, Valério RM (1999). Cerebellar hemorrhage after supratentorial surgery for treatment of epilepsy: report of three cases. *Neurosurgery*.

[7] Göbet F, Heidecke V, Hube R, Reichel H, Held A, Hein W (1999). Cerebellar hemorrhage as an early complication of spine surgery. *Zeitschrift fur Orthopadie und Ihre Grenzgebiete*.

[8] Friedman JA, Ecker RD, Piepgras DG, Duke DA (2002). Cerebellar hemorrhage after spinal surgery: report of two cases and literature review. *Neurosurgery*.

[9] Brockmann MA, Nowak G, Reusche E, Russlies M, Petersen D (2005). Zebra sign: cerebellar bleeding pattern characteristic of cerebrospinal fluid loss. *Journal of Neurosurgery*.

[10] Khalatbari MR, Khalatbari I, Moharamzad Y (2012). Intracranial hemorrhage following lumbar spine surgery. *European Spine Journal*.

[11] Sciubba DM, Kretzer RM, Wang PP (2005). Acute intracranial subdural hematoma following a lumbar CSF leak caused by spine surgery. *Spine*.

[12] Karaeminoğulları O (2005). Remote cerebellar hemorrhage after a spinal surgery complicated by dural tear: case report and literature review. *Neurosurgery*.

[13] Morofuji Y, Tsunoda K, Hiu T (2009). Remote cerebellar hemorrhage after cervical spinal surgery: two case reports and literature review. *Neurological Surgery*.

